# Humidity and Gravimetric Equivalency Adjustments for Nephelometer-Based Particulate Matter Measurements of Emissions from Solid Biomass Fuel Use in Cookstoves

**DOI:** 10.3390/ijerph110606400

**Published:** 2014-06-19

**Authors:** Sutyajeet Soneja, Chen Chen, James M. Tielsch, Joanne Katz, Scott L. Zeger, William Checkley, Frank C. Curriero, Patrick N. Breysse

**Affiliations:** 1 Department of Environmental Health Sciences, Bloomberg School of Public Health, Johns Hopkins University, Baltimore, MD 21205, USA; E-Mail: ssoneja@jhsph.edu; 2 Program in Global Disease Epidemiology and Control, Department of International Health, Bloomberg School of Public Health, Johns Hopkins University, Baltimore, MD 21205, USA; E-Mails: chechen@jhsph.edu (C.C.); jkatz@jhsph.edu (J.K.); wcheckl1@jhmi.edu (W.C.); 3 Department of Global Health, School of Public Health and Health Services, George Washington University, Washington, DC 20037, USA; E-Mail: jtielsch@email.gwu.edu; 4 Department of Biostatistics, Bloomberg School of Public Health, Johns Hopkins University, Baltimore, MD 21205, USA; E-Mail: szeger@jhsph.edu; 5 Division of Pulmonary and Critical Care, School of Medicine, Johns Hopkins University, Baltimore, MD 21205, USA; 6 Department of Epidemiology, Bloomberg School of Public Health, Johns Hopkins University, Baltimore, MD 21205, USA; E-Mail: fcurrier@jhsph.edu

**Keywords:** nephelometer, particulate matter, humidity adjustment, gravimetric equivalent, pDR, low resource environment, biomass burning, cookstove, indoor air quality

## Abstract

Great uncertainty exists around indoor biomass burning exposure-disease relationships due to lack of detailed exposure data in large health outcome studies. Passive nephelometers can be used to estimate high particulate matter (PM) concentrations during cooking in low resource environments. Since passive nephelometers do not have a collection filter they are not subject to sampler overload. Nephelometric concentration readings can be biased due to particle growth in high humid environments and differences in compositional and size dependent aerosol characteristics. This paper explores relative humidity (RH) and gravimetric equivalency adjustment approaches to be used for the pDR-1000 used to assess indoor PM concentrations for a cookstove intervention trial in Nepal. Three approaches to humidity adjustment performed equivalently (similar root mean squared error). For gravimetric conversion, the new linear regression equation with log-transformed variables performed better than the traditional linear equation. In addition, gravimetric conversion equations utilizing a spline or quadratic term were examined. We propose a humidity adjustment equation encompassing the entire RH range instead of adjusting for RH above an arbitrary 60% threshold. Furthermore, we propose new integrated RH and gravimetric conversion methods because they have one response variable (gravimetric PM_2.5_ concentration), do not contain an RH threshold, and is straightforward.

## 1. Introduction

Assessment of exposure-disease relationships related to use of solid biomass fuels (wood, dried animal manure, and crop residue) for cooking and other household energy needs in the developing world has become a top priority [1]. With approximately 3 billion people using solid biomass fuels, large scale interventions using new, more efficient cooking technologies are being conducted to reduce adverse health effects associated with solid biomass fuel use [1,2]. The success of these efforts will hinge in part on the degree to which new stove technology reduces pollutants and the corresponding reduction in disease. Uncertainties in the exposure disease relationships make designing interventions difficult since the exposure reduction targets are not known. Currently, great uncertainty exists around the exposure-disease relationships due to lack of detailed exposure data in large health outcome studies. Recent reviews have highlighted the need to conduct more detailed exposure assessments in health studies of household air pollution (HAP) and cookstoves [2,3].

Particulate matter (PM) is a principal emission from biomass combustion and significant contributor to morbidity and mortality. PM measurement technology available for cookstove exposure assessments were designed for use in developed countries where PM concentrations are typically 2 to 3 orders of magnitude lower [2]. Filter-based integrated gravimetric samplers are difficult to use in biomass burning cookstove settings because the high PM concentrations require low flow rates and short sample times to minimize sampler overload. Furthermore, limitations in battery life, specifically for the air pump component of the sampler, make collecting samples for a day or more problematic in low resource settings where access to electricity can be limited. Passive nephelometric devices for measuring PM have several advantages over filter-based integrated sampling methods. They can be used for longer periods on a single charge and do not require a filter, so overloading is less of a problem.

Nephelometers, however, have a number of important limitations. Since light scattering is an indirect measure of PM concentration, the devices need to be calibrated against a gravimetric standard. Nephelometers come pre-calibrated using standard fine dust test aerosol (Arizona road dust) [4]. Since the particle size distribution and composition of cookstove PM is different from Arizona road dust, the instrument response needs to be adjusted to account for differences in PM characteristics. In addition, since many aerosols are hygroscopic and will increase in size at high humidity, nephelometer response needs to be adjusted for humidity effects [5,6,7,8].

The purpose of this paper is to describe humidity and gravimetric adjustment approaches for the DataRAM pDR-1000 (Thermo Scientific, Franklin, MA, USA) used to assess indoor PM concentrations in a large cookstove intervention trial in Nepal (registered as NCT00786877). This nephelometer measures airborne PM passively, providing a direct, continuous readout with data storage for subsequent analyses [4]. The pDR uses light (wavelength of 880nm) with a scattering coefficient range (1.5 × 10^−6^ to 0.6 m^−1^) to illuminate particulate and estimate light scattered [4,5]. Calibration by the manufacturer is performed using a gravimetric standard, International Organization for Standardization Fine test dust [4]. Given the working principle of the nephelometer and results from previous studies, concentration readings are subject to possible bias as a result of differences in aerosol characteristics and sampling conditions [5,6,7]. Specifically, adjustments for relative humidity during measurement and conversion to gravimetric equivalents need to be performed.

## 2. Background

### 2.1. Adjusting Recorded PM Data for Relative Humidity

Changes in particle size distribution with increasing humidity can result in an overestimation of PM concentration [8]. To account for the influence of relative humidity (RH) in nephelometric measurements, a correction factor is typically applied. This correction factor can be calculated as the ratio of the humidity influenced PM nephelometric concentration to the humidity independent PM concentration. Dividing the nephelometric measurements by this correction factor will adjust for the PM-humidity bias. Since PM composition and size may impact how humidity is accounted for, it is preferable to estimate the correction factor in laboratory settings where particulate size and composition can be carefully varied and characterized [9,10]. However, when laboratory studies are not feasible, or aerosol characteristics are unknown or highly variable, the correction factor can be estimated using statistical models and field sampling data. Published approaches to assessing humidity bias adjustment have used either measurement from paired filter-based monitors (corrected for humidity by equilibration of filters in humidity controlled weigh rooms prior to weighing) or from nephelometers with a heater attached to the inlet [7,11]. Richards *et al.* utilized heaters attached to the inlet of nephelometers to eliminate the influence on PM measurements from high RH levels [6], while Wu *et al.* questioned the ability of the heater attached to the inlet to remove the influence from high RH [8]. Since filter-based monitors incorporate a humidity equilibration step in the weighing process, paired gravimetric and nephelometer samples are more commonly used to estimate humidity correction factors.

Utilizing data from different sources to account for RH, previous studies demonstrated two main humidity adjustment equations to estimate the correction factor. An empirical adjustment equation derived from experimental data of Laulainen *et al.* [12] fits well with data from several studies [11,13]:

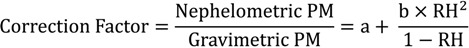
(1)
where *a* is equal to 1 and *b* is equal to 0.25, as reported by Chakrabarti *et al.* [11]. No discussion was given regarding the determination of these parameters.

Another adjustment derived from simple linear regression by Richards *et al.* [6], was proven to fit well in data presented by Wu *et al.* [7]:

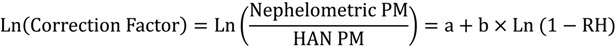
(2)
where *a* and *b* are empirically determined parameters by linear regression, and the denominator is humidity adjusted nephelometric PM concentration (HAN PM) measured by a nephelometer with a heated inlet.

These equations apply for RH values above 60%, which is believed to be the threshold at which nephelometers begin to significantly overestimate particle concentrations as a result of RH increase [7,10,11].

### 2.2. Converting Recorded PM Data to Gravimetric Equivalents

To estimate equivalent mass concentrations, passive nephelometer readings are usually adjusted to account for differences in the manufacturer calibration aerosol and the real-world aerosol being sampled using co-located gravimetric samples. Regression models are typically used to relate gravimetric PM measurements and humidity adjusted nephelometric PM [7,11,14,15,16]:

Gravimetric PM = a + b × HAN PM(3)

where *a* and *b* are empirically determined parameters by linear regression, and HAN PM is humidity adjusted nephelometric PM. The humidity adjusted nephelometric measurement is generally an overestimate of the gravimetric equivalent PM concentration by a factor ranging from slightly greater than one to three [7,16,17,18,19]. None of the previously published gravimetric correction factors have been determined for the large range of concentrations seen in indoor biomass cookstove environments where long-term time-weighted average concentrations can span more than three orders of magnitude [7,14,16,17,18,19].

## 3. Methods

### 3.1. Study Overview

The study site in rural, southern Nepal was established by the Department of International Health at Johns Hopkins Bloomberg School of Public Health. Under the broad effort known as the Nepal Nutrition Intervention Project—Sarlahi (NNIPS) [20], several studies are underway, one of which includes a cookstove intervention trial designed to assess indoor PM exposure to adverse health effects. Located in Sarlahi District, Nepal, the entire NNIPS site consists of 32 areas referred to as Village Development Committees (VDCs), four of which are currently participating in the cookstove trial. Sarlahi is a rural area located in the Terai region of southern Nepal (on the border with Bihar State in India) and is representative of southern Nepal and most of northern India with elevation approximately 200 m above sea level [20]. For our study, we utilized data from the parent cookstove trial as well as a substudy within this trial (known as the Simulated Cooking Test). Both studies utilized the pDR-1000 and HOBO U10 Temperature and Humidity Data Logger (Onset Computer Corporation, Pocasset, MA, USA), recording data in 10-second intervals.

### 3.2. Parent Cookstove Trial

Data from air quality studies measuring indoor PM associated with traditional cookstove emissions were collected at this study site. Cooking with traditional stoves, comprised of clay mud, bricks, rice husk, and cow dung, are common in this area of Nepal [21]. The cookstove trial includes 2854 homes, each with at least two 24-hour indoor PM concentration measurements using the pDR-1000. Data from this study included co-located pDR-1000 and humidity measurements.

### 3.3. Simulated Cooking Test

Co-located pDR-1000, gravimetric PM, and humidity samples were collected during simulated cooking events in a mock house and in homes participating in the parent cookstove trial. Mock house sampling was conducted in a house built to represent a typical house in this region, determined from data collected during the parent cookstove trial. The mock house consisted of a 1-room floor plan with 1-window and door, with the ability to close and open these features. Housing material consisted of bamboo with mud, logs, and tree branches, while roof material consisted of half tile and half thatch/grass. House dimensions were: length 3.85 m, width 4.65 m, ground to the lowest point of the roof 1.8 m, ground to the apex of the roof 2.7 m, window 0.6 m by 0.6 m (located on the back wall of the house), and door frame 1.28 m width by 1.64 m height (located on the front wall). Both the window and door had a hinged wood-framed metal panel attached to it that allowed for opening/closing according to the prescribed test conditions. In addition, a traditional mud-based cookstove with two openings was built inside according to typical practices for stove construction, located on the floor of the back wall. Co-located PM and humidity samples were collected during cooking activities with different fuel types using a standard cooking protocol.

To assess pDR-1000 performance, a modified version of the Water Boiling Test (WBT) 3.0 was used [22]. A standard cooking session was simulated by bringing two pots of water (5 L in each) to a rolling boil from ambient temperature (this requires approximately 30 min). The fire was then extinguished. Passive PM sampling was initiated 30 min prior to the cook test and continued for an additional two hours post-fire, while gravimetric sampling took place only during the active flame period. For this analysis, Passive PM data were examined only during the active flame period. In addition, pDR-1000s were zeroed before every test using procedures recommended by the manufacturer. A limited number of co-located pDR samples were collected in the mock house to assess precision, which resulted in an average precision of 11%. This value is in good agreement with a previously published study finding precision to be from 3% to 13% [5].

Gravimetric PM was collected with a PM_2.5_ inlet (BGI, Waltham, MA, USA) on Teflon filters (37 mm 2.0 µm pore PTFE Membrane Filter w/ PMP ring Pel Life Sciences, Ann Arbor, MI, USA) using a personal sampling pump (5400 BGI Inc., Waltham, MA, USA) at a flow rate of 4 L/min. Flow rates were calibrated before and after sampling using a Drycal Flowmeter (DC Light BIOS Intl., Butler, NJ, USA). Filters were pre and post-weighed in a temperature and humidity controlled weighing room using a XP2U Microbalance (Mettler Toledo, Columbus, OH, USA) located at the Johns Hopkins Bloomberg School of Public Health.

Pre-weighed Teflon filters were loaded into polypropylene filter cassettes (SKC Inc., Eighty Four, PA, USA), along with filter pads (Pall Life Sciences, Ann Arbor MI) and 37 mm drain discs (model No. 230800 Air Diagnostics and Engineering, Harrison, ME, USA) at the Harioun clinic in a field-developed clean box to minimize contamination during assembly while in Nepal. The sampling equipment in the mock house was located 1 m from the stove and 1.8 m above the floor. After sampling, the filter cassettes were placed in plastic bags until returned to the United States for post weighing. For quality control purposes, duplicate gravimetric samples were collected for 10% of the test runs, resulting in an average duplicate precision of 12% and relative SD of 9%. Limit of detection was calculated to be 5 μg and all filter weights were blank corrected (2 μg).

Upon completion of testing in the mock house, the same test protocol was performed in 50 occupied homes that were randomly selected from the 2854 households involved in the parent cookstove trial from two of the four cookstove VDC’s. Due to equipment failure during sampling, 10 homes were excluded from analysis. Homes chosen reflected typical housing based on preliminary data from the NNIPS cookstove intervention trial study (i.e. have 1 window/door, similar in size and composition to the mock house). Eligible homes met the criteria of only using cookstoves for personal food production, agreeing to not have any other sources of combustion ongoing in the house during testing, and were confirmed to ensure that no occupants used tobacco. Testing was conducted midday in order to minimize interference to participants’ daily routines as well as smoke infiltration from surrounding homes’ cooking activities. Fuel type was allowed to vary based on cooking preferences of the home. The type of fuel was observed for subsequent analysis of results by fuel type.

### 3.4. Statistical Analysis

Data from different fuel types, including wood, crop waste, and wood with anything else (*i.e.*, dung, crop waste, or a mixture), window/door status (all closed *vs.* anything open), home type (mock *vs.* occupied), and kitchen size were incorporated into the analysis to evaluate different adjustment equations. The influence of these variables on humidity and gravimetric adjustment was evaluated by adding these variables into regression models, and then assessed for inclusion in the final model based upon significance of their p-values and other standard regression model evaluation procedures [23].

Root mean square errors (RMSEs) of predicted values in each equation were calculated in order to assess fit and accuracy of prediction of the equations to our data. RMSE was estimated by incorporating all data points into the equation when parameters were not estimated from our data. For equations where parameters were estimated from our data, the RMSEs were calculated by conducting leave-one-out cross validation. In detail, one data point was removed out of 65 total points as a test point, whereupon parameters for the equation were trained (*i.e.*, re-estimated) with the remaining 64 data points and used to predict a value for the test point. This process was repeated 65 times for each data point; at each iteration the squared residual error (difference between the predicted and “true” test point) was calculated and then averaged. The square root of these mean squared errors (RMSE) provides a measure of prediction accuracy on the original scale of the data.

Cross validation was also conducted to assess overall fit and prediction accuracy for the two-step quality control methods. After initially removing one data point randomly, the humidity adjustment equation was trained with the remaining data points. Parameters of the gravimetric conversion equation were then ascertained with the same data points adjusted for RH. Applying the test value sequentially on both equations, we were able to calculate the cross validated RMSE.

All statistical analyses were performed in the R Statistical Computing Environment (Version 3.0.2; 25 September 2013, Vienna, Austria). Packages used for data analysis and graphics creation were chron [24] and ggplot2 [25].

## 4. Results and Discussion

### 4.1. Humidity Adjustment

Three separate humidity adjustment equations derived from Equations (1) or (2) (referred to as 1*a*, 1*b*, and 2*a* in Table 1) were examined. Equation (1*a*) was derived from Equation (1), with the parameters given in Chakrabarti’s 2004 publication [11]. For Equation (1*b*), we fitted Equation (1) with our data collected in the mock house and occupied homes, where the correction factor was calculated as the ratio of average pDR-1000 PM concentration and the corresponding gravimetric PM_2.5_ concentration. Similarly, we fitted Equation (2) with our data to derive Equation (2*a*). Table 1 presents the regression parameters for the three equations. The range of nephelometric PM concentration data was observed to be from ~600 μg/m^3^ to ~66,000 μg/m^3^.

**Figure 1:**
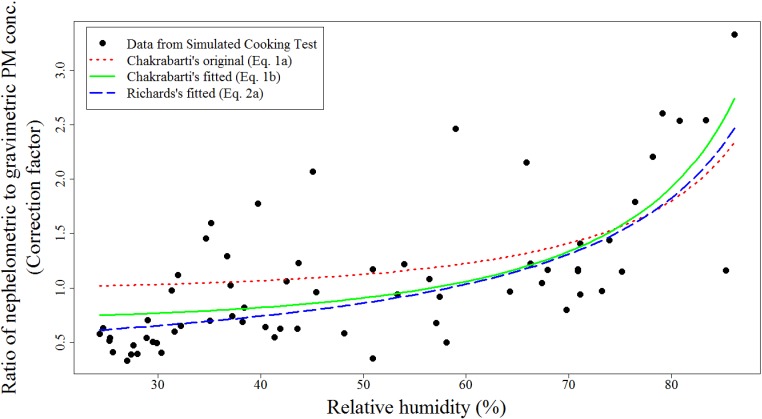
Humidity adjustment Equations (1*a*), (1*b*), and (2*a*) displayed with data collected during cooking for both the mock house and occupied homes.

As shown in Figure 1, all humidity adjustment equations agree well with each other and provide a reasonable description of the correction factor versus humidity results. According to the RMSE estimated by cross validation presented in Table 1, all equations performed equally with a suggestion that Equation (2*a*) provided a slightly better fit. Adjustment with and without a 60% humidity threshold did not make a large difference in fit, with Equations (1*b*) and (2*a*) performing slightly better without a threshold and Equation (1*a*) performing better with a threshold. Although Equation (1*b*) and Equation (2*a*) were based on different approaches, both demonstrated similar fitted lines and RMSE after incorporating experimental data, suggesting that as long as experimental data were incorporated, applying any of the two humidity adjustment equations will not dramatically affect the results.

Below 40% RH, most data points have correction factors less than 1.0, with all correction factors less than 1.0 for RH less than 30%, indicating underestimation of PM concentration by nephelometers at very low humidity. Similar trends were identified in Chakrabarti *et al.* where this underestimation was noted to start at 20% RH [11]. This observed trend could be due to PM size reduction during low RH levels. In addition, filter weights were determined after filter equilibration at 35% RH supporting the observation of pDR-1000 underestimation relative to gravimetric at low humidity. It should be noted that a substantial increase from a correction factor above 1.0 was not observed until 70% RH in our data, 75% RH in Chakrabarti’s 2004 data, and 50% and 65% RH in Day’s 2001 data [11,13]. These results, combined with our observation of a negative bias at low humidity, suggest that the choice of 60% RH threshold for humidity is not well supported.

**Table 1:** Summary of regression parameters and RMSE for the three humidity adjustment equations.

Equation	Parameter a (95% CI)	Parameter b (95% CI)	RMSE ^†^ with Threshold	RMSE ^†^ without Threshold
Equation (1*a*) *	1 ^**#**^	0.25 ^**#**^	0.506	0.514
Equation (1*b*) **	0.72 (0.65, 0.79)	0.38 (0.33, 0.44)	0.521	0.495
Equation (2*a*) ***	−0.72 (−0.82, −0.62)	−0.82 (−0.93, −0.71)	0.515	0.490

Notes: ^**#**^ Based on original Chakrabarti equation, no confidence intervals were provided [11]; * Equation (1*a*) = Chakrabarti’s original humidity adjustment equation; ** Equation (1*b*) = Chakrabarti’s humidity adjustment equation fitted with simulated cooking test data; *** Equation (2*a*) = Richards’s humidity adjustment equation fitted with simulated cooking test data; **^†^** RMSE is unitless.

**Figure 2:**
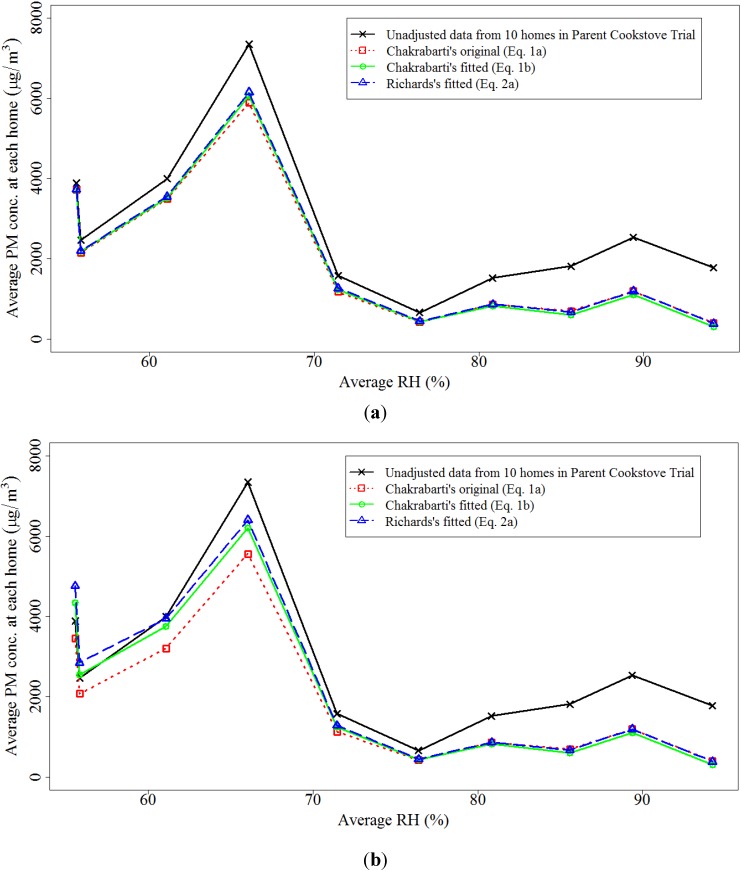
Average PM concentrations of 10 homes adjusted with three humidity adjustment equations (**a**) with a RH threshold and (**b**) without a RH threshold.

To illustrate the influence of the three humidity adjustment Equations (1*a*), (1*b*), and (2*a*) using real-world samples, we compared 24-hour PM concentration data adjusted for RH collected using pDR-1000’s in 10 homes within the parent cookstove trial. As shown in Figure 2, the means of humidity adjusted nephelometric PM concentrations are similar using the three adjustment equations across all household average RH values, while Equation (1*b*) and Equation (2*a*) without a threshold lead to higher means of adjusted values in the lower RH range. These results illustrate that humidity adjustment without threshold provides better compensation for the negative bias at low humidity, which is consistent with the RMSE values reported in Table 1.

An additional comparison of the three humidity adjustment equations was conducted by applying the humidity adjustment Equations to 24-hour pDR data from all 2854 homes. There was no statistically significant difference in the mean PM concentrations after humidity adjustment for the equations (Equations (1*a*), (1*b*), and (2*a*)) that incorporated a 60% RH threshold (ANOVA *p* = 0.7). Mean PM concentrations were statistically different when the same equations incorporating no RH threshold were used (Kruskal-Wallis *p* < 0.0001). However, when only considering equations trained with our data (Equations (1*b*) and (2*a*)), we identified no significant difference with or without a RH threshold. These results are consistent with the relationship demonstrated in Figure 1.

### 4.2. Gravimetric Conversion

**Figure 3:**
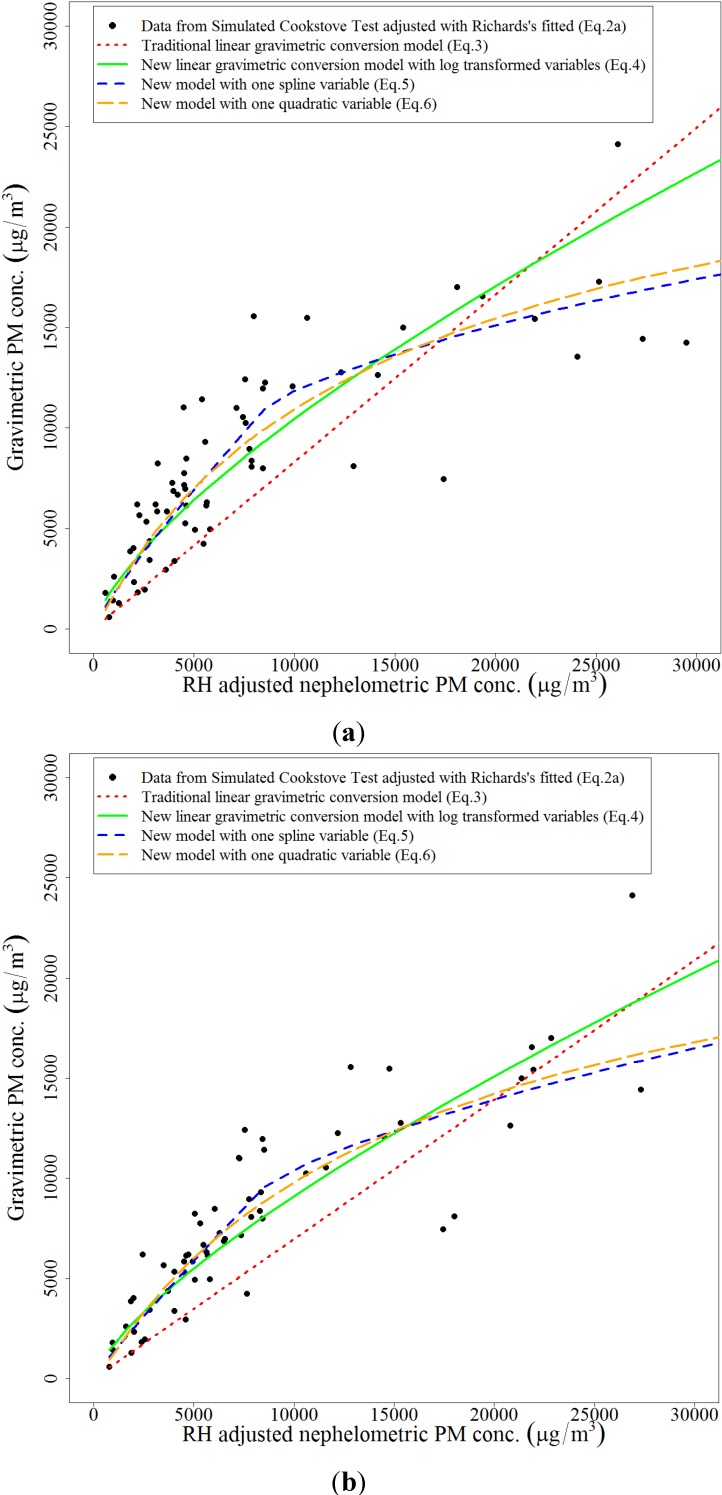
Linear, linear with log transformed variables, linear with spline variable, and linear with quadratic variable Equations for gravimetric conversion based on nephelometric PM concentrations adjusted with RH adjustment Equation (2*a*). (**a**) RH adjustment with 60% threshold; (**b**) RH adjustment without 60% threshold.

Once the humidity adjustments were made, we compared the adjusted pDR-1000 average results to the co-located gravimetric PM_2.5_ concentrations using four equations (Equation (3) previously discussed, along with Equations (4–6) discussed below). From the linear regression equation (Equation (3)), we estimated that gravimetric PM concentration is equal to humidity adjusted nephelometric PM times the slope coefficient, while forcing the Equation through the origin. Our Simulated Cooking Test data had average concentrations that spanned more than three orders of magnitude. In addition, our data in Figure 3 indicated a non-linear relationship between humidity adjusted nephelometric PM and gravimetric PM. Therefore, we evaluated the linear relationship between the natural log of both gravimetric PM and humidity adjusted nephelometric PM (Equation (4)):

Ln(Gravimetric PM) = a + b × Ln(HAN PM)(4)

where *a* and *b* are empirically determined parameters by linear regression of log transformed experimental data. We also evaluated a slightly altered version of Equation (4). Recognizing that the gravimetric to nephelometric PM relationship had a turning point, we included a spline term which yielded the following Equation:

ln⁡(Gravimetric PM_2.5_) = a + b × ln⁡(HAN PM)+ c × (ln ⁡(HAN PM)− d) (5)

where d represents the spline point and c is equal to zero when the natural log of nephelometric PM is smaller than d. A range of possible spline points was determined by observing the relationship between gravimetric and humidity adjusted PM concentrations. This range spanned 7.5 to 12 for natural log of nephelometric PM concentration. Utilizing increments of tenths, values within this range were fitted into the equation. Resulting cross validated RMSEs were compared to choose the best spline point d. Another equation we evaluated to assess the nonlinear gravimetric to nephelometric relationship was in quadratic form:

ln⁡(Gravimetric PM_2.5_) = a + b × ln⁡(Nephelometric PM) + c× (ln⁡(Nephelometric PM))^2^ (6)

where parameters were empirically determined by regression.

Table 2 and Figure 3 summarize the regression parameters and RMSE values for four gravimetric conversion equations (Equations (3‒6), respectively) with data adjusted using three humidity adjustment approaches (Equations (1*a*), (1*b*) and (2*a*) with and without a 60% RH threshold). Based upon the RMSE values, the new linear gravimetric conversion equation with log transformed variables fits better to RH adjusted data (both with and without a threshold) compared to the traditional linear approach. Moreover, the new linear gravimetric conversion Equation utilizing a quadratic variable (Equation (6)) fits the best to RH adjusted data (both with and without a threshold) compared to all equations, but only slightly better than the equation utilizing a spline variable (Equation (5)). The curves in Figure 3a and Figure 3b are consistent with the RMSE values estimated via cross validation. The range of gravimetric PM concentration in our study was observed to be from ~600 μg/m^3^ to ~26,000 μg/m^3^, while the upper limit for most other published studies [16] did not exceed 600 μg/m^3^. This broader range of PM concentration relative to other studies is one possible explanation for this observed improved performance when utilizing either a log transformed, spline, or quadratic approach during gravimetric conversion. The RMSE values in Table 2 are relatively similar by approach, suggesting that utilizing the three RH adjustment equations, with or without a 60% RH threshold, provide similar gravimetric equivalent estimates with a preference towards a spline or quadratic approach.

**Table 2:** Summary of gravimetric equivalency conversion for the three humidity adjusted results (with and without a 60% RH threshold) utilizing a linear, linear with log transformed variables, linear with log transformed and spline variable, and linear with log transformed and quadratic variable Equations.

Equation Type	Coefficient and RMSE Values	With Threshold			Without Threshold		
		Equation 1*a*	Equation 1*b*	Equation 2*a*	Equation 1*a*	Equation 1*b*	Equation 2*a*
Linear eqn. (Equation (3))	a	0	0	0	0	0	0
	b	0.848	0.845	0.831	0.892	0.757	0.696
	RMSE **^†^**	3927	4002	4005	3956	3982	3955
Linear eqn. w/log transformed variables (Equation 4)	a	2.726	2.750	2.753	2.723	2.510	2.395
	b	0.711	0.707	0.706	0.715	0.724	0.730
	RMSE **^†^**	2889	2977	2969	2932	3001	2990
Linear eqn. w/log transformed and spline variables (Equation 5)	a	0.859 *	0.872 *	0.822 *	0.565 *	0.921 *	0.868 *
	b	0.949	0.948	0.953	0.995	0.921	0.917
	c	−4.051	−0.411	−0.416	−0.430	−0.471	−0.502
	d	8.4	8.4	8.4	8.2	8.9	9.1
	RMSE **^†^**	2703	2768	2773	2742	2650	2620
Linear eqn. w/log transformed and quadratic variables (Equation 6)	a	−4.867 *	−4.945	−4.951	−4.994 *	−6.049	−6.607
	b	2.502	2.527	2.522	2.544	2.722	2.809
	c	−0.105	−0.106	−0.106	−0.107	−0.115	−0.119
	RMSE **^†^**	2682	2750	2759	2715	2652	2619

Notes: * Not significantly different from 0 (*p* > 0.05); **^†^** RMSE is μg/m^3^.

### 4.3. Combined Quality Control Method vs. the Two-step Method

To simplify the two-step process, we propose combining the humidity and gravimetric equivalency adjustments into one equation. Based on the humidity adjustment work by Richards *et al*. and the linear gravimetric conversion Equation with log transformed variables (Equations (2) and (4), respectively), a combined humidity and gravimetric equivalency adjustment equation was derived. This derivation yielded:

ln⁡(Gravimetric PM_2.5_) = 3.102 + 0.701 × ln⁡(1 − RH) + 0.717 × In (Nephelometric PM)⁡ (7)

where parameters were determined based on data from the simulated cooking test.

Unlike the traditional two-step quality control method, which has RH adjustment and gravimetric conversion done separately, the 60% RH threshold is not used for this new combined quality control method (Equation (7)). Given the issues associated with the arbitrariness of 60% RH threshold and the possible negative bias at low humidities discussed earlier, excluding it when conducting adjustments improves the accuracy of the adjusted data.

Another combined humidity and gravimetric equivalency adjustment equation was derived from Equations (2) and (5), respectively. This derivation yielded inclusion of a spline term, thus resolving into:

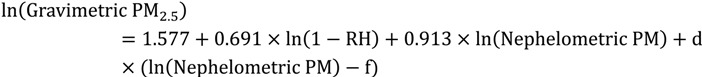
(8)
where f is 8.1. This was determined by examining the relationship between the gravimetric and unadjusted nephelometric PM, and comparing cross validated RMSEs of equations with different spline points as described above. This relationship was further evaluated via the use of RMSE to finalize f. It should be noted that when the log nephelometric PM concentration is >f, the parameter d = −0.254; otherwise, the parameter d=0, thus resulting in the spline term being excluded.

An additional combined approach was developed from Equations (2) and (6), respectively. This approach resulted in a quadratic term, which yielded:

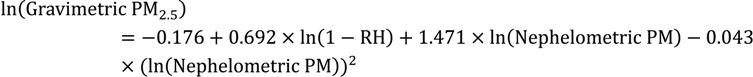
(9)
Parameters from Equations (8) and (9) were determined from the simulated cooking test.

To evaluate the combined approaches (Equations (7‒9)) we compared their performance to the eighteen combinations of RH adjustment and gravimetric conversion for the traditional two-step quality control method (linear gravimetric equivalency equations eliminated) (Table 3). As shown in Table 3, regardless of which RH adjustment Equation used, there is minimal impact on the prediction accuracy of the two-step approach. The two-step quality control combinations with and without the 60% RH threshold have similar accuracy in prediction when both quality control methods are applied, thus further supporting the rational of not including a threshold.

According to Table 3, the combined Equation approach utilizing a spline (Equation (8)) provides the best RMSE amongst the three combined approaches. The combined quality control methods have RMSE values that are higher than the lowest reported RMSE values for the two-step approaches. However, it is important to consider potential explanations that could result in these varying RMSE values. The approach of the two step quality control methods utilize the same reference value twice, which could lead to overfitting, thus a lower RMSE value.

An important point to note is that at lower PM concentration and RH ranges, the quality control methods suggest that nephelometric data may underestimate gravimetric concentration. This could be the result of our broad range in PM concentration values collected during biomass fuel combustion, which has not been well studied utilizing the pDR-1000. It should also be noted that making adjustments below nephelometric PM concentration of 600 μg/m^3^ could lead to uncertainty due to the absence of data below this value.

**Table 3:** Summary of different overall pDR-1000 adjustment approaches comparing the RSE values.

Quality Control Method Type	Approach Number	RMSE (μg/m^3^)
Combined Approach (1)	1	3066
Combined Approach—spline (2)	2	3007
Combined Approach—quadratic (3)	3	3243
Equations (1*a*), (1*b*) & (2*a*) (without threshold) + Equation (4)	4, 5, 6	2922, 2959, 2962
Equations (1*a*), (1*b*) & (2*a*) (without threshold) + Equation (5)	7, 8, 9	2641, 2600, 2593
Equations (1*a*), (1*b*) & (2*a*) (without threshold) + Equation (6)	10, 11, 12	2696, 2628, 2607
Equations (1*a*), (1*b*) & (2*a*) (with threshold) + Equation (4)	13, 14, 15	2925, 2948, 2948
Equations (1*a*), (1*b*) & (2*a*) (with threshold) + Equation (5)	16, 17, 18	2652, 2687, 2689
Equations (1*a*), (1*b*) & (2*a*) (with threshold) + Equation (6)	19, 20, 21	2716, 2736, 2744

Further analysis explored the impact of fuel type (wood, crop waste, mix of wood and other), home type (occupied vs. mock), kitchen size, and window/door status on Equation (7). These variables were not statistically significant and therefore were not included.

Even though the two-step combinations provide for a better fit according to the RMSE values, the new combined quality control method holds several advantages for future applications. First, the combined quality control method involves only one reference value, excluding the possibility of over-fitting with having to use multiple reference values. In addition, it is quicker and easier to perform an adjustment using an integrated approach (as done in Equation (7)) than to have to perform multiple steps as is done in the two-step approach. Furthermore, integration of a spline or quadratic term requires additional examination of data in order to properly assess whether their use is warranted. For our data, the equation with the spline term (Equation (8)) provides for the best RMSE across all one-step equations. Given this along with the advantages of using a one-step method, Equation (8) is believed to be the best approach with all that has been presented.

## 5. Conclusions

In this paper we have explored a range of humidity and gravimetric equivalency adjustment approaches. Three approaches (Equations (1*a*), (1*b*), and (2*a*)) to humidity adjustment all performed equivalently (similar RMSE values). Previous research suggests that humidity overestimation bias is observed when humidity exceeds 50% to 75% [11,13]. Our results suggest that an overestimation bias is close to the 75% RH value. In addition, an underestimation bias exists at very low RH (<30%). As a result, we have proposed humidity adjustment equations that encompass the entire RH range. Furthermore, the humidity adjustment using the equation by Chakrabarti *et al.* (Equation (1*a*)), which was derived by sampling ambient PM in Southern California, performed similarly to humidity adjustments calculated using cookstove PM samples collected in Nepal. This suggests that humidity adjustments do not vary widely based on the characteristics of the PM being sampled.

Given the wide range of concentration in our study, the new linear gravimetric conversion equations with log transformed variables performed better than the traditional linear regression gravimetric conversion equation. Furthermore, gravimetric conversion equations incorporating a spline or quadratic term provided for the best fit amongst equations with log transformed variables. The two-step quality control combinations utilizing the new linear gravimetric conversion equation with log transformed variables also have better accuracy in prediction than those utilizing the traditional linear gravimetric conversion equation (data not shown). Our PM concentration range, collected during biomass burning, was much broader than other published studies, which could explain why the new linear gravimetric conversion equations with log transformed variables and either a spline or quadratic term demonstrated a better fit. Moreover, given the higher concentration range observed in our study, the adjustments proposed should be applied for nephelometric PM concentration ranging from ~600 μg/m^3^ to ~66,000 μg/m^3^.

In general, utilization of the traditional two-step method is less preferred than the integrated RH and gravimetric conversion methods presented in this paper (Equations (7), (8), and (9)) for a variety of reasons. Principally, the integrated method is preferred because it only involves one response variable (gravimetric PM_2.5_ concentration), avoids overfitting, does not contain a RH threshold, and is relatively quick and straightforward. For our data, we recommend using the combined method that includes a spline term (Equation (8)) for quality control, based on the RMSE value. In order to achieve the best adjustment, we recommend readers to assess their own data to choose which combined quality control method to utilize, using the approaches outlined in this paper. Providing an approach to determine humidity corrected gravimetric equivalent PM_2.5_ concentrations will allow systematic comparison exposure response relationships in health studies using the pDR-1000.
